# Building mitotic chromosomes

**DOI:** 10.1016/j.ceb.2010.09.009

**Published:** 2011-02

**Authors:** Shinya Ohta, Laura Wood, Jimi-Carlo Bukowski-Wills, Juri Rappsilber, William C Earnshaw

**Affiliations:** Wellcome Trust Centre for Cell Biology, University of Edinburgh, Michael Swann Building, King's Buildings, Mayfield Road, Edinburgh EH9 3JR, Scotland, UK

## Abstract

Mitotic chromosomes are the iconic structures into which the genome is packaged to ensure its accurate segregation during mitosis. Although they have appeared on countless journal cover illustrations, there remains no consensus on how the chromatin fiber is packaged during mitosis. In fact, work in recent years has both added to existing controversies and sparked new ones. By contrast, there has been very significant progress in determining the protein composition of isolated mitotic chromosomes. Here, we discuss recent studies of chromosome organization and provide an in depth description of the latest proteomics studies, which have at last provided us with a definitive proteome for vertebrate chromosomes.

## Chromosome anatomy and formation

Mitotic chromosome structure has fascinated cell biologists since the 19th century, but we still know relatively little about the composition and topology of chromosomes. We know even less about the activities responsible for the remarkable structural transformation that occurs when the chromatin of the interphase nucleus adopts the characteristic ‘X-shaped’ morphology as metazoan cells enter mitosis.

**Figure 1:**
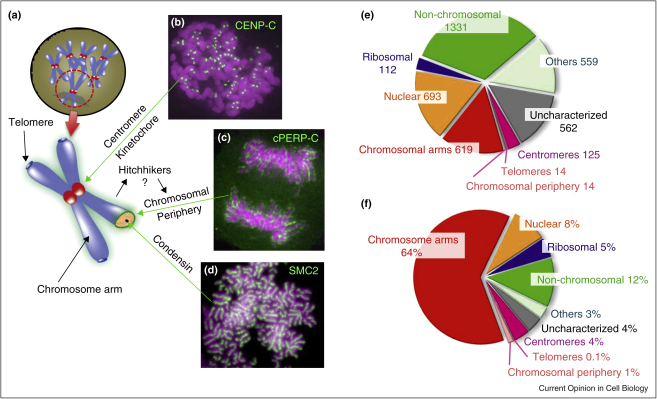
Functional subdomains in mitotic chromosomes (**a**), include, (**b**) Centromeres, Telomeres, (**c**) the Chromosome periphery and (**d**) chromosome arms. **(e)** The 9 classes of proteins found in chromosomes. **(f)** Estimated percentages of total chromosomal protein mass in the major classes of proteins [32^••^].

Mitotic chromosomes have four structural/functional domains: centromeres, telomeres, the periphery, and arm chromatin (Figure 1a–d). Each domain has a characteristic protein composition. The centromere and its associated kinetochore together comprise an elaborate structure, with over 120 constituents described to date. They bind spindle microtubules and direct chromosome segregation in mitosis [1,2]. The protein composition of telomeres is relatively simpler [3,4]. Telomeres play an essential role in protecting chromosome ends and preventing chromosome fusion events. The chromosome periphery (perichromosomal layer) may act like a skin protecting the chromosome surface [5–8]. Its components are enriched in ribosomal and nucleolar proteins [9–11]. Many may simply be ‘hitchhikers’—proteins that bind to chromosomes in the cytoplasm following nuclear envelope breakdown and serve no essential function during mitosis. Others appear to function during chromosome segregation, as discussed below.

Experiments by Hirano and co-workers identified the condensin complex as a factor essential for mitotic chromosome formation in cell-free Xenopus egg extracts [12]. This complex is distributed along the axial region of the chromosome arms (Figure 1d). Key condensin components are the SMC proteins [13], which have roles in many types of chromosome transactions. Condensin, cohesin, and SMC proteins are reviewed regularly [14–17].

Condensin is required for successful completion of mitosis, but not for mitotic chromosome formation *in vivo* [18–21]. Condensin is important for the timing of chromosome condensation [19,20], the elastic properties of chromosomes [22] and centromeres [23], the segregation of rDNA in yeast [24,25], dosage compensation in *C. elegans* [26], and chromosome integrity during anaphase [20,21]. However, something else must be the driving factor behind mitotic chromosome formation. Dephosphorylation of a target termed RCA (**r**egulator of **c**hromosome **a**rchitecture) by the Repo-Man:PP1 (**p**rotein **p**hosphatase 1) holoenzyme correlates with a dramatic loss of chromosome organization during anaphase in cells lacking condensin [27].

The molecular identity of RCA has yet to be determined. RCA could be a specific non-histone protein, or a combination of histone post-translational modifications. A recent study identified H3T3**p**hK4**m**e2R8**m**e2 (termed the PMM mark) [28] as specific for mitotic chromosomes. The PMM mark is not essential for mitotic chromosome formation, but could form part of a more complex histone modification pattern that promotes mitotic chromosome formation. Ultimately, the identity of RCA and mechanism of condensin action in mitotic chromosome formation remain mysterious.

Another protein previously linked with mitotic chromosome formation is DNA topoisomerase II (topo II) [29–31], one of the most abundant non-histone proteins of mitotic chromosomes [32^••^]. However, RNAi and genetic knockouts subsequently revealed that topo II is dispensable for mitotic chromosome formation [33–35]. A recent *in vitro* biophysical analysis has suggested that DNA entanglements have a role in determining the physical properties of mitotic chromosome arms [36^••^] (for review see [37]). Thus, topo II could have an important influence on the behavior of chromosomes as they respond to forces within the mitotic spindle (see also [38,39]).

## Chromosome topology

Much effort has been spent in trying to confirm or refute a visionary model proposed by Laemmli—that mitotic chromosomes consist of chromatin loops constrained by interactions with a scaffolding of non-histone proteins [40,41]. Enthusiasm for a non-histone scaffold has waxed and waned over the years [42,43]. Microscopy studies have tended to support the role of some sort of axial determinants of mitotic chromosome structure [44–47]. However, *in vitro* studies suggest that if chromatin loops are constrained by proteins, those loops must be relatively small [48]. Furthermore, more recent examination of isolated chromosomes has suggested that order is minimal within the chromosome, with chromatin folding in the paired sister chromatids showing little, if any reproducibility [49^•^]. This contrasts markedly with previous studies showing that chromosome arms can adopt a helical conformation with mirror symmetry [45,50].

There has been scant progress in recent years in understanding the higher order packing of chromatin in mitotic chromosomes. Since the first proposal of the solenoid model of nucleosome packing [51], it has been generally assumed that mitotic chromosomes consist of a hierarchy of higher order packaged chromatin fibers. Indeed, detailed analysis of budding yeast chromatin compaction *in vivo* suggested that most of the chromatin has a level of compaction consistent with the solenoid model [52]. By contrast, a recent electron cryomicroscopy study failed to find any evidence for 30 nm chromatin fibers in isolated mitotic chromosomes [53^••^]. Those authors suggested that the chromatin is so tightly packed that interactions between nucleosomes of adjacent fibers compete with those between nucleosomes on the same fibers [53^••^]. This could destabilize the solenoid, creating a densely packed amorphous mass of nucleosomes referred to as a ‘polymer melt’. An earlier EM tomography study looking at chromosomes assembled *in vitro* in Xenopus egg extracts had also failed to observe prominent 30 nm fibers, instead visualizing nucleosomes clustered into a network of 30–40 nm domains [54]. There is no doubt that the 30 nm solenoid exists *in vitro*, but its role *in vivo* may continue to be debated over the coming years (reviewed in [55]).

Because chromosomal substructures fall into a ‘resolution gap’ between the electron microscope and conventional light microscopes, technological advances have a significant impact on our understanding of chromosome structure. In one recent study, coherent x-ray diffraction was used to examine isolated chromosomes [56^•^]. In this pioneering study, the chromosomes appeared to have a denser axial region, contrasting with the results from electron cryomicroscopy [53^••^]. A second approach that is just beginning to be applied to mitotic chromosomes is super-resolution light microscopy, an area in which remarkable technical advances have been made in recent years. PALM (photoactivation localization microscopy) has recently been used to analyze the kinetochore, mapping the distribution of CENP-A relative to other inner kinetochore proteins at 37 nm resolution [57^•^]. If this or a related technology can in the future be applied to ‘native’ chromosomes, it may finally enable the path of fiber folding to be traced in intact chromosomes.

It is clear that mitotic chromosomes continue to offer mysteries and challenges, even at the most basic levels of their structure.

## Chromosome composition

Isolated mitotic chromosomes are roughly 2:1 protein to nucleic acid on a mass basis [58,59]. About half of this protein is histone, but the remainder is often lumped together under the not-very informative term ‘non-histone proteins’. In recent years, significant strides have been made in the identification and characterization of these non-histone proteins.

Purification of mitotic chromosomes is not straightforward, as many cytoplasmic proteins bind tightly to the highly charged chromosomes after nuclear envelope breakdown. These proteins cannot be separated from the chromosomes without harsh chemical extractions, so it can be extremely difficult to define what is and is not a *bona fide* chromosomal protein. This issue, which we term the ‘hitchhiker problem’ [32] has been addressed by the Fukui lab [60^•^], but a solution remains elusive because conventional fractionation procedures such as centrifugation cannot separate chromosomes from contaminants that adhere to them physically (Figure 1a).

The first two proteomic analyses of mitotic chromosomes [61,62] tried to avoid the ‘hitchhiker problem’ by characterizing chromosome scaffolds produced by digesting isolated chromosomes with micrococcal nuclease and extracting >90% of the proteins with 2 M NaCl [63]. The first report identified 62 proteins, including a novel protein of the chromosome periphery, NGB/CRFG, but was bedeviled by the presence of numerous mitochondrial contaminants [61]. A follow-up project identified 79 proteins in chromosome scaffolds [62], including the novel proteins—Borealin [64] and CENP-V [65] as well as two other proteins of the chromosome periphery. Another study characterized proteins from Xenopus egg extract that bound to added sperm chromatin [66^•^]. This report did not give a lengthy description of the entire proteome identified, but instead focused on characterization of the novel kinetochore protein Bod1.

A particularly thorough set of studies of the mitotic chromosome proteome has been carried out by the Fukui laboratory [9,60,67]. They identified ∼250 proteins in isolated mitotic chromosomes, ∼100 of which are likely to be specific chromosomal proteins. Their subsequent work has focused on functional analysis of several proteins found at the chromosome periphery: nucleophosmin, nucleolin and **r**egulator of **r**ibosome **s**ynthesis 1 (RRS1). Surprisingly, all three were found to be necessary for timely and efficient alignment of the chromosomes during prometaphase [68–70]. The underlying mechanisms are unknown.

The analysis of centromeres and telomeres by proteomics has been a particular challenge, as they are differentiated regions of the single long chromosomal DNA molecule, rather than independent structures in their own right. A particularly elegant solution was taken to the isolation of telomeres. This involved the use of DNA hybridization to fish out the TTAGGG sequences that characterize human telomeres, a procedure that the authors termed PICh (proteomics of isolated chromatin segments) [71^••^]. That study found 98 proteins common to telomeres from telomerase positive and ALT cell lines (which maintain telomeres by recombination rather than telomerase activity). These included the components of the shelterin complex, known to be involved in chromosome end protection and maintenance [3,4]. The study also found a similar number of proteins specific to each class of telomeres [71^••^]. One surprise was the finding of several orphan receptors associated with ALT telomeres, which the authors proposed might have a role in promoting telomere association with PML bodies [71^••^].

Proteomic characterization of kinetochores has involved affinity purification of proteins that associate with kinetochore components such as CENP-A [72–74] or CENP-S [75]. One recent study reporting the isolation of entire budding yeast minichromosomes led to the discovery of a PP1 regulatory subunit, Fin1, associated with the kinetochore [76^••^]. Fin1 is involved with regulation of the spindle checkpoint. Kinetochores have been extensively reviewed elsewhere [1,2], so these studies will not be discussed further here.

## Multi-classifier combinatorial proteomics (MCCP) of mitotic chromosomes

One recent study used a procedure developed by the Laemmli lab [77] to isolate mitotic chromosomes from chicken DT40 cells for proteomic analysis, capitalizing on quantitative proteomics software developed by the Mann lab [78^•^]. This work yielded a list of ∼4000 proteins (Figure 1e). Known and predicted chromosomal proteins comprised 72% of the total protein mass present (Figure 1f), indicating that the purification procedure was quite successful. Of the ∼4000 proteins, >550 were previously uncharacterized.

One attempt to solve the ‘hitchhiker problem’ described above was to apply **s**table **i**sotope **l**abeling by **a**mino acids in **c**ell culture (SILAC) [79] to chromosomes subjected to a variety of different analytical procedures. This technique accurately compares protein ratios between samples by determining ratios of individual peptides distinguished by ^13^C/^15^N and ^12^C/^14^N, using cultures grown in heavy and light medium, respectively. SILAC was used to determine the percentage of each protein in isolated chromosomes relative to an equal mass of cytosol and to measure the ability of cytosolic proteins to bind stably to isolated chromosomes. SILAC was also combined with genetic ablation of key proteins to look at dependency relationships governing the chromosomal association of various proteins and protein complexes. The data set generated from each such experiment was the ratio of heavy-to-light peptides for each protein. This quantitatively reflected the distribution of each protein in the samples being compared, and enabled the proteome to be sorted as a ranked list. Each sorted list was termed a classifier.

**Figure 2:**
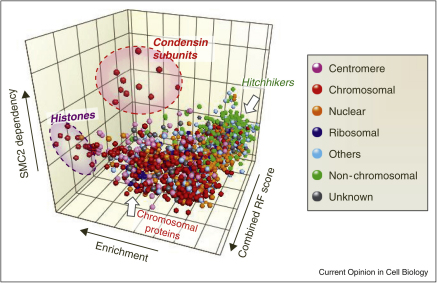
Combining classifiers in 3-dimensions increases specificity. In this case the axes plot Enrichment (ratio of each protein in chromosomes versus that in an equal protein mass of cytoplasm) versus SMC2 dependency (amount of each protein in wild type chromosomes divided by its amount in chromosomes from SMC2-depleted cells) versus the Combined random forest score (calculated by combining all proteomic classifiers with nearest neighbor analysis and quantitative bioinformatic analysis of protein domains) [32^••^]. Core histones and condensin subunits cluster in the analysis.

This analysis was initially unsatisfying, as no classifier could reliably distinguish chromosomal from non-chromosomal proteins. This problem was solved by combining the classifiers. Since each classifier is simply a list of values, it can be used to define the axis of a graph. Using the classifiers mentioned above, one could plot for all proteins in the data set their enrichment in chromosomes versus their ability to exchange onto chromosomes versus their dependency on a protein such as condensin subunit SMC2 (a related plot is shown in Figure 2). Plotting parameters that seem to be independent of one another in this way yielded powerful insights.

The following example shows how this analysis can work. In a three-dimensional plot such as that of Figure 2, one can use the *k*-nearest neighbor algorithm (*k*-NN—a type of machine learning [80]) to ask for every uncharacterized protein in the three-dimensional space which of its *k* nearest neighbors is known to be chromosomal. This generates a list in which uncharacterized proteins are ranked according to the quality of their neighborhood. Varying *k* enables one to alter the effective ‘resolution’ of the analysis (for example identifying proteins likely to be centromeric rather than simply chromosomal). As a further step, the neighborhood values for each individual experiment and the original data can be input into another machine learning algorithm such as Random Forest analysis [81], which can be trained to separate chromosomal from non-chromosomal proteins using proteins of known behavior. The efficiency of this approach was tested by tagging 50 novel/uncharacterized proteins with GFP and observing their localization in mitosis (12 novel centromere proteins; 7 novel periphery proteins, 11 novel bulk chromatin proteins). Of the 50 tagged proteins 44 (88%) localized in mitosis as predicted. This enabled the prediction that among the ∼550 uncharacterized proteins of the chromosome proteome, 97 new centromere-associated proteins; 46 new chromosome periphery proteins, and 90 new bulk chromatin proteins remain to be identified [32^••^].

The MCCP approach also enables the experimenter to reveal subtle relationships between characterized and uncharacterized proteins. The experimental framework underpinning this, cluster-heatmap analysis, has been used for many years to analyze microarray data and compare samples generated from different cell types or cell types exposed to differing experimental conditions. What has been recently realized is that this analysis need not be limited to microarray data. In fact, any combination of quantitative data can be used. To date, classifiers used in cluster-heatmap analysis have included phenotypic profiling of cell cycle defects [82,83^•^], SILAC ratios from proteomic experiments [32^••^,84,85], quantitative analysis of protein interaction data [86] and quantitative analysis of protein localization data [87^•^]. One powerful outcome of this analysis is that it can allow the prediction of protein function for proteins whose primary sequence is uninformative [32^••^].

When combined with genetics, the MCCP approach also allows one to study and even identify protein complexes in their ‘native environment’ by analyzing the entire mitotic chromosome fraction without requiring that protein complexes be available in soluble form. In a demonstration of this approach, genetic ablation of Ska3 was found to result in the loss of the Ska, APC/C, and RanBP2/RanGAP1 complexes from chromosomes, and all subunits of all complexes behaved in an identical manner [32^••^]. Thus this approach can be used to deduce the composition of functional protein complexes and dependency relationships between them without the need for biochemical fractionation.

## Towards a molecular model of the chromosome

Now that comprehensive lists of proteins are available, development of the next generation of models for the molecular organization of chromosomes will require two further advances: (1) a way to determine the copy numbers of all of the various chromosomal constituents and (2) a method to map protein–protein contacts between all chromosomal proteins. The first of these is now becoming a reality. Starting with the budding yeast, where there is known to be a single Cse4/CENP-A-positive nucleosome in the kinetochore of each of the 16 chromosomes, it has been possible to use GFP-tagged proteins and quantitative fluorescence methods to determine the copy numbers for a number of kinetochore components [88]. This analysis was extended to *S. pombe* [89], and most recently to the kinetochores of chicken DT40 cells [90^•^]. This analysis is quite laborious, but recent improvements in the analysis of proteomic data have permitted initial estimation of copy numbers for all kinetochore proteins in DT40 cells [32^••^]. These show a remarkable agreement with the values from fluorescence measurements, and when the method is further developed, rigorous quantitation of all protein components of mitotic chromosomes will be possible.

Mapping all protein interactions within entire chromosomes sounds far fetched, but is approaching the realm of possibility. This can in principle be done by protein–protein cross-linking followed by proteomic identification of all cross-linked peptides. The method has been successfully applied to the outer kinetochore-associated Ndc80 complex [91], and more recently to the considerably larger complex of RNA polymerase II holoenzyme bound to the initiation factor TIIF [92^•^]. Significant technical advances are required before this could be applied to an entire mitotic chromosome. Nonetheless, it now appears possible that within the next few years, the molecular architecture of mitotic chromosomes will be understood at a previously unimagined level of detail.

## Note added in proof

Since this review was written a second study has been published using super-resolution microscopy to study mitotic chromosome structure under conditions of minimal disruption. This study reports that Drosophila embryo mitotic chromatin is largely composed of ∼70 nm fibers. Relating observations made under super-resolution conditions to conventional images will be a challenge for the future [93].

## References and recommended reading

Papers of particular interest, published within the annual period of review, have been highlighted as:

• of special interest
•• of outstanding interest
